# Cigarette Smoking Increased Risk of Overall Mortality in Patients With Non-alcoholic Fatty Liver Disease: A Nationwide Population-Based Cohort Study

**DOI:** 10.3389/fmed.2020.604919

**Published:** 2020-12-07

**Authors:** Phunchai Charatcharoenwitthaya, Khemajira Karaketklang, Wichai Aekplakorn

**Affiliations:** ^1^Division of Gastroenterology, Department of Medicine, Faculty of Medicine Siriraj Hospital, Mahidol University, Bangkok, Thailand; ^2^Department of Community Medicine, Faculty of Medicine Ramathibodi Hospital, Mahidol University, Bangkok, Thailand

**Keywords:** cigarette smoking, alcohol, mortality, non-alcoholic fatty liver disease, gender

## Abstract

**Background:** The evidence suggests a detrimental effect of cigarette smoking on the progression of chronic liver disease. However, the impact of cigarette smoking on mortality among patients with non-alcoholic fatty liver disease (NAFLD) remain unclear.

**Methods:** We used the National Health Examination Survey data collected during 2008–2009 to link the National Death Index to follow-up respondent survival. Diagnosis of NAFLD was based on a lipid accumulation product in participants without significant alcohol use or other liver diseases.

**Results:** During 64,116 person-years of follow-up, 928 of 7,529 participants with NAFLD died, and the cumulative all-cause mortality was 14.5 per 1,000 person-years. In a Cox regression model adjusted for age, body mass index, alcohol intake, exercise, comorbidities, lipid profiles, and handgrip strength, current smoking increased the risk of mortality by 109% (adjusted hazard ratio (aHR): 2.09, 95% confidence interval [CI]: 1.18–3.71) compared with never smoker status in women, but showed only a trend toward harm among men (aHR: 1.41, 95% CI: 0.96–2.08). After controlling for potential confounders, smoking ≥10 pack-years continued to show a significant harmful effect on all-cause mortality among women (aHR: 5.40, 95% CI: 2.19–13.4), but not in men. Among women who drink alcohol ≥10 grams per day, current smoking (aHR: 13.8, 95% CI: 1.66–145) and smoking ≥10 pack-years (aHR: 310, 95% CI: 78–1,296) also significantly increased risk of death.

**Conclusion:** This nationwide population-based study highlight a detrimental effect of cigarette smoking on mortality, with a similar but more definite association in women than in men with NAFLD.

## Introduction

Non-alcoholic fatty liver disease (NAFLD) is a growing global health problem that affects almost a quarter of the world's population (1). NAFLD-related liver complications are predicted to become the most common indication for liver transplantation within the next decade (2). Notably, a large body of clinical evidence indicates that NAFLD is also associated with an increased risk of other extrahepatic manifestations, such as cardiovascular disease and extrahepatic cancers, which are the predominant causes of mortality in patients with NAFLD (3–6). Since NAFLD causes a considerable health burden worldwide, it is important to identify modifiable risk factors and develop prevention strategies.

Cigarette smoking is a known major risk factor for developing chronic diseases, such as chronic obstructive pulmonary disease, cardiovascular disease, and several malignancies (7–9). The risk of death from these conditions increases with increasing exposure to cigarette smoking, as measured by the number of cigarettes smoked daily and the duration of smoking (10). There is also growing evidence that cigarette smoking has negative association with the prognosis of chronic liver diseases (11–16). Cigarette smoking was demonstrated to be associated with a dose-dependent relationship with the stage of liver fibrosis in patients with NAFLD via its effect on insulin resistance (11).

Patients with advanced liver fibrosis are believed to have the highest risk of progressing to end-stage liver disease. A recent meta-analysis of 4,428 NAFLD patients showed fibrosis stage to be the histological feature independently associated with liver-related events, liver-related mortality, and overall mortality (17). The relationship between fatal clinical outcomes and lifestyle risk factors, mainly moderate alcohol consumption, has been established (18). However, little is known about the effect of cigarette smoking on long-term prognosis among persons with NAFLD in the general population. Accordingly, this study was designed to evaluate the effect of cigarette smoking on overall mortality in patients with NAFLD after adjustment for important potential confounders using patient data derived from a nationwide population-based cohort.

## Materials and Methods

### Study Population

Study participants were enrolled from the Fourth Thai National Health Examination Survey (NHES-IV), which is a nationally representative survey that employs a complex multistage, stratified strategy to sample Thai civilian, non-institutionalized population (19). The NHES-IV cohort included 21,960 persons aged ≥15 years that were recruited during August 2008 to March 2009. In the present study, 19,181 persons aged ≥18 years were included in the analysis.

### Data Collection and Measurements

All information was collected in face-to-face interviews conducted by research nurses using standardized questions. Smoking status was classified as never smoker, former smoker, or current smoker. Participants who smoked <100 cigarettes during their lifetime were classified as never smokers. Current smokers were defined as those who currently smoke and who reported having smoked more than 100 cigarettes in their lifetime. Former smokers were defined as those who reported having smoked >100 cigarettes in their lifetime, but who no longer smoked at all at the time of the health checkup examination. Cigarette smoking was also quantified as pack-years, which was defined as the average number of packs per day multiplied by the number of years as a smoker. Alcohol consumption was calculated using self-reported questionnaire items relating to the frequency and amount of alcohol use per day over the 12-months period preceding the examination. The weekly frequency of physical activities was assessed by the Global Physical Activity Questionnaire version 2.

Blood pressure was measured using a standard automatic blood pressure monitor. Weight, height, and waist circumference (WC) were measured using standard procedures, and body mass index (BMI) was calculated as weight in kilograms divided by height in meters squared. Obesity was defined as BMI ≥25 kg/m^2^, and central obesity was defined as a WC of >90 cm for men and >80 cm for women, following the Asian-specific criteria (20). Metabolic syndrome was defined according to the harmonizing criteria (21). Handgrip strength was measured by digital dynamometer.

Blood samples were obtained from participants in the morning after an overnight fast. The samples were transferred for determination of fasting plasma glucose using an enzymatic hexokinase method. Total cholesterol, low-density lipoprotein cholesterol (LDL-C), triglycerides, and high-density lipoprotein cholesterol (HDL-C) were measured by homogeneous enzymatic colorimetric methods. The non-HDL-C and total cholesterol to HDL-C ratio were calculated to estimate cardiovascular risk.

### Definition of NAFLD

**Figure 1:**
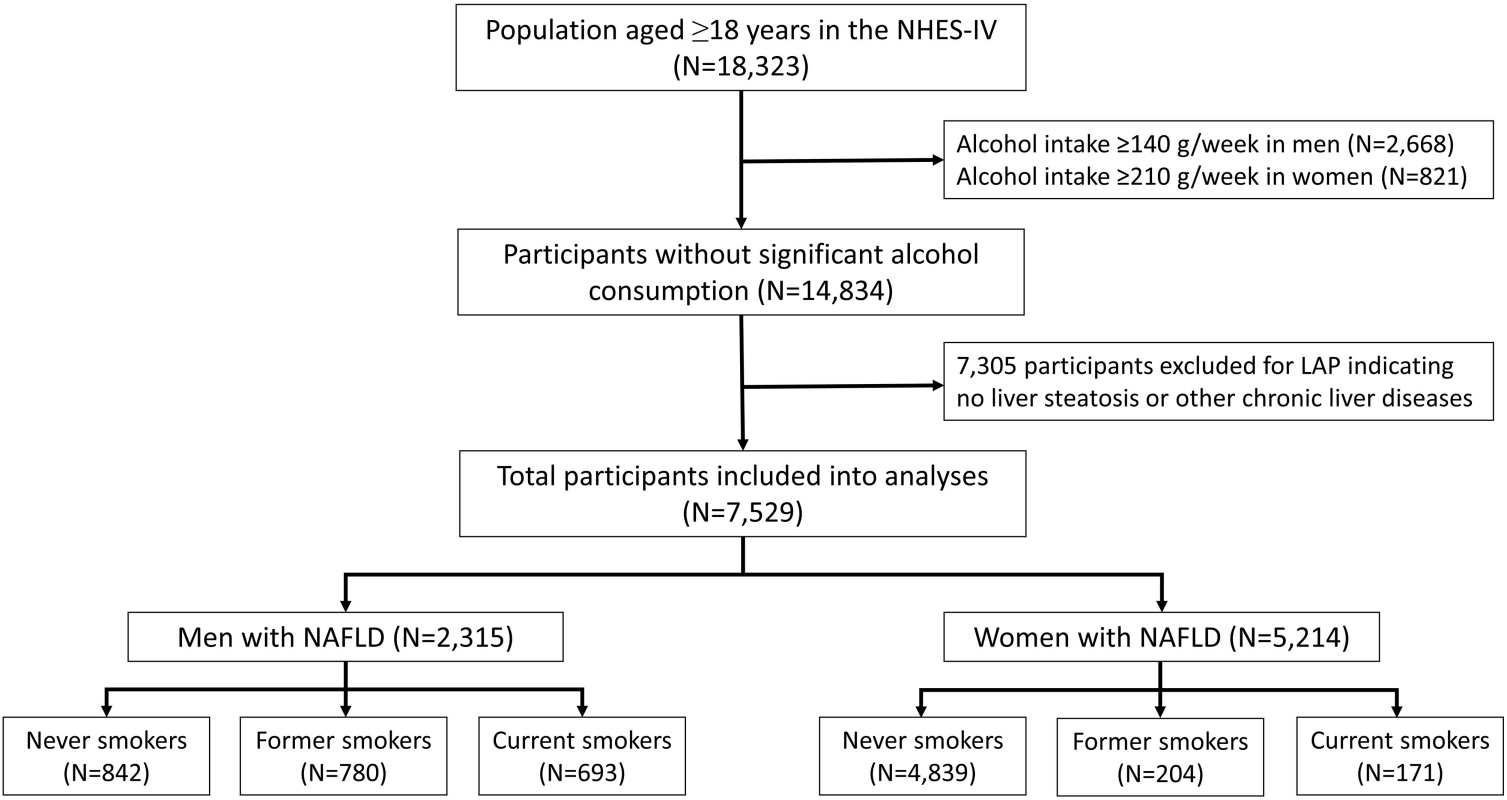
Flow diagram of the study protocol. NHES-IV, National Health Examination Survey IV; LAP, lipid accumulation product; NAFLD, non-alcoholic fatty liver disease.

To identify patients with NAFLD, NHES participants with the following conditions were excluded: excessive alcohol consumption (defined as >210 g/week for men, and >140 g/week for women) or any other possible causes of chronic liver disease (Figure 1). Lipid accumulation product (LAP) was the parameter that was then used to identify NAFLD among the remaining subjects (22, 23). LAP is a non-invasive method that predicts the presence of NAFLD based on the patient's WC and triglyceride concentration that is calculated using the formula: [WC (cm) – 65] × triglyceride concentration (mmol/L)] for men, and [WC (cm) – 58] × triglyceride concentration (mmol/L)] for women. This model was reported to be a valid assessment for establishing NAFLD in general population. Using ultrasound as a reference, hepatic steatosis can be predicted by LAP with an area under the curve value of 0.843 (95% confidence interval [CI]: 0.837–0.849) in men, and 0.887 (95% CI: 0.882–0.892) in women (23). In the present study, participants were presumed to have NAFLD if they had a LAP score of ≥30.5 in men, and ≥23.0 in women (23).

### Mortality Follow-Up

Follow-up and mortality data were obtained by linking the NHES data to the National Civil Registration and Vital Statistics System, Ministry of Interior, which is the most reliable database source of mortality data in Thailand. Information regarding vital status was obtained from the date of NHES-IV survey participation to 31 December 2019, or to the date of death. The study was carried out in accordance with the Declaration of Helsinki and was approved by the Institutional Review Board. Written informed consent was obtained from all participants during the initial assessment.

### Statistical Analysis

The analysis took into account the complex survey design, since all of the estimates were weighted according to the inverse probability of being sampled based on the registered Thai population. Chi-square test and one-way analysis of variance (ANOVA) were used to compare the baseline characteristics of study participants among the smoking status categories. *Post hoc* multiple comparison analysis was performed with Bonferroni correction. The survival analysis of participants with NAFLD was performed using unadjusted Kaplan-Meier method, and log-rank test was used to compare survival distribution between groups.

Cox proportional hazard regression models were constructed to estimate adjusted hazard ratios (aHRs) of overall mortality based on smoking status and pack-years. Models were initially adjusted for age, and then further adjusted for BMI, alcohol intake, regular exercise, and medical conditions (metabolic syndrome, diabetes, hypertension, and history of cardiovascular and cerebrovascular diseases). To account for other potential confounders, a fully adjusted analysis was performed, which included handgrip strength and LDL-C, HDL-C, and triglyceride levels. All analyses were performed using STATA version 14.0 (StataCorp LP, College Station, Texas, USA). A two-sided *p*-value of <0.05 was considered statistically significant.

## Results

### Population Characteristics

After excluding participants with significant alcohol use and other possible causes of chronic liver disease, 14,834 participants were eligible for inclusion (Figure 1). Of those, 2,315 men (38.3%) and 5,214 (59.4%) women had a LAP score of ≥30.5 and ≥23, respectively, and those 7,529 participants were classified as having NAFLD. The proportions of never, former, and current smokers based on the self-report were 36.4, 33.7, and 29.9% in men, respectively, and 92.8, 3.9, and 3.3% in women, respectively. Among the current smokers, the median pack-year of cigarette smoking was 15 (range: 8–25.6) in men, and 8.5 (range: 4.2–15) in women.

Among men, current smokers were more likely to be younger, alcohol drinkers, have higher measurements for glucose, triglycerides, total cholesterol to HDL-C ratio, and handgrip strength and lower HDL-C, and less likely to have hypertension and history of cerebrovascular disease compared with never smokers (Table 1). Former smokers were more likely to be older, alcohol drinkers, have hypertension and history of cerebrovascular disease, and have lower measurements for BMI, WC, and handgrip strength compared with never smokers. In women, both former and current smokers were more likely to be older and have lower measurements for BMI and handgrip strength compared with never smokers (Table 2). Current smokers were more likely to have a lower mean HDL-C, whereas former smokers were more likely to have metabolic syndrome and higher triglyceride concentrations compared with never smokers.

**Table 1:** Baseline characteristics of male study participants with NAFLD by smoking status.

**Characteristics**	**Smoking status**				***p*-value**	**Multiple comparison**
	**Overall**	**Never smoker (a)**	**Former smoker (b)**	**Current smoker (c)**		
Number (%)	2,315 (100%)	842 (36.4%)	780 (33.7%)	693 (29.9%)		
Age (years)	49.1 ± 13.2	47.7 ± 13.2	55.0 ± 12.5	46.5 ± 12.3	**<0.001**	a ≠ b ≠ c
Body mass index (kg/m^2^)	26.6 ± 3.8	27.0 ± 3.8	26.4 ± 3.6	26.3 ± 3.8	**<0.001**	a ≠ b, a ≠ c
Waist circumference (cm)	90.7 ± 9.2	91.8 ± 9.3	91.0 ± 9.2	89.6 ± 9.0	**<0.001**	a ≠ b ≠ c
Obesity, *n* (%)	1,354 (62.8%)	541 (66.9%)	432 (60.3%)	381 (60.5%)	0.053	
Alcohol intake, *n* (%)	747 (38.7%)	219 (29.7%)	242 (41.0%)	286 (45.5%)	**<0.001**	a ≠ b, a ≠ c
Regular exercise, *n* (%)	1,729 (78.7%)	619 (77.8%)	615 (81.8%)	495 (77.6%)	0.506	
Cardiovascular disease, *n* (%)	93 (2.2%)	31 (1.5%)	44 (4.2%)	18 (1.5%)	**0.031**	a ≠ b, b ≠ c
Cerebrovascular disease, *n* (%)	83 (2.6%)	30 (4.0%)	42 (3.6%)	11 (0.6%)	**0.011**	a ≠ c, b ≠ c
Metabolic syndrome, *n* (%)	1,548 (59.2%)	582 (62.2%)	556 (60.2%)	410 (55.7%)	0.273	
Diabetes mellitus, *n* (%)	421 (11.2%)	148 (9.4%)	171 (13.2%)	102 (11.7%)	0.073	
Hypertension, *n* (%)	1,131 (33.6%)	421 (34.7%)	447 (41.6%)	263 (27.2%)	**<0.001**	a ≠ b ≠ c
Systolic blood pressure (mmHg)	130 ± 18	131 ± 19	133 ± 18	127 ± 15	**<0.001**	a ≠ c, b ≠ c
Diastolic blood pressure (mmHg)	80 ± 10	81 ± 10	81 ± 10	80 ± 10	**<0.001**	a ≠ c, b ≠ c
Glucose (mg/dL)	96 ± 31	96 ± 31	98 ± 32	95 ± 29	**<0.001**	a ≠ c, b ≠ c
Total cholesterol (mg/dL)	220 ± 44	221 ± 42	215 ± 45	223 ± 45	0.179	
LDL-C (mg/dL)	133 ± 40	135 ± 40	129 ± 38	134 ± 42	0.184	
HDL-C (mg/dL)	40 ± 8.9	40 ± 8.4	40 ± 8.3	38 ± 9.6	**<0.001**	a ≠ c, b ≠ c
Non-HDL-C (mg/dL)	180 ± 42	180 ± 39	175 ± 42	184 ± 43	**0.031**	b ≠ c
Total cholesterol to HDL-C ratio	5.7 ± 1.3	5.6 ± 1.2	5.5 ± 1.2	6.0 ± 1.4	**<0.001**	a ≠ c, b ≠ c
Triglycerides (mg/dL)	245 ± 130	226 ± 115	246 ± 132	261 ± 140	**<0.001**	a ≠ c, b ≠ c
Handgrip strength (kg)	38.6 ± 8.6	38.7 ± 9.1	36.8 ± 8.2	39.7 ± 8.1	**<0.001**	a ≠ b ≠ c

*A p-value <0.05 indicates statistical significance*.

*Data presented as number and percentage or mean ± standard deviation. LDL-C, low-density lipoprotein cholesterol; HDL-C, high-density lipoprotein cholesterol; NAFLD, nonalcoholic fatty liver disease*.

*Chi-square test and one-way analysis of variance (ANOVA) were used to compare the baseline characteristics of study participants among the smoking status categories. Post hoc multiple comparison analysis was performed with Bonferroni correction*.

*a ≠ b ≠ c: Statistically significant difference among never smoker (a), former smoker (b), and current smoker (c)*.

*a ≠ b, a ≠ c: Statistically significant difference between never smoker (a) and former smoker (b), and between never smoker (a) and current smoker (c); however, there was no significant difference between former smoker (b) and current smoker (c)*.

*b ≠ c: Statistically significant difference between former smoker (b) and current smoker (c)*.

*a ≠ b, b ≠ c: Statistically significant difference between never smoker (a) and former smoker (b), and between former smoker (b) and current smoker (c); however, there was no significant difference between never smoker (a) and current smoker (c)*.

**Table 2:** Baseline characteristics of female study participants with NAFLD by smoking status.

**Characteristics**	**Smoking status**				***p*-value**	**Multiple comparison**
	**Overall**	**Never smoker (a)**	**Former smoker (b)**	**Current smoker (c)**		
Number (%)	5,214 (100%)	4,839 (92.8%)	204 (3.9%)	171 (3.3%)		
Age (years)	49.6 ± 13.6	49.2 ± 13.5	59.9 ± 12.5	55.7 ± 13.1	**<0.001**	a ≠ b ≠ c
Body mass index (kg/m^2^)	26.8 ± 4.3	26.9 ± 4.3	25.4 ± 4.5	24.8 ± 3.7	**<0.001**	a ≠ b, a ≠ c
Waist circumference (cm)	85.9 ± 9.3	86.0 ± 9.3	84.7 ± 9.1	82.9 ± 8.8	**<0.001**	a ≠ c
Obesity, *n* (%)	3,181 (64.3%)	3,004 (65.1%)	101 (55.4%)	76 (41.6%)	**0.001**	a ≠ c
Alcohol intake, *n* (%)	619 (15.6%)	563 (15.6%)	28 (17.1%)	28 (15.1%)	0.879	
Regular exercise, *n* (%)	3,743 (81.1%)	3,490 (81.1%)	131 (75.7%)	122 (83.9%)	0.391	
Cardiovascular disease, *n* (%)	169 (2.2%)	153 (2.2%)	9 (2.4%)	7 (1.9%)	0.873	
Cerebrovascular disease, *n* (%)	127 (2.0%)	118 (2.0%)	7 (2.0%)	2 (0.6%)	0.135	
Metabolic syndrome, *n* (%)	2,228 (36.4%)	2,056 (36.4%)	106 (58.3%)	66 (37.7%)	**0.010**	a ≠ b
Diabetes mellitus, *n* (%)	917 (13.2%)	846 (13.2%)	44 (16.1%)	27 (11.4%)	0.678	
Hypertension, *n* (%)	2,330 (33.1%)	2,154 (32.7%)	104 (45.7%)	72 (35.1%)	0.171	
Systolic blood pressure (mmHg)	127 ± 19	127 ± 18	132 ± 18	125 ± 21	**0.009**	a ≠ b, b ≠ c
Diastolic blood pressure (mmHg)	77 ± 10	77 ± 10	79 ± 10	75 ± 11	0.053	
Glucose (mg/dL)	95 ± 34	95 ± 34	104 ± 38	99 ± 28	0.101	
Total cholesterol (mg/dL)	219 ± 45	219 ± 45	228 ± 49	220 ± 47	0.634	
LDL-C (mg/dL)	138 ± 41	138 ± 41	144 ± 45	140 ± 41	0.961	
HDL-C (mg/dL)	45 ± 10	46 ± 10	44 ± 10	44 ± 11	**0.023**	a ≠ c
Non-HDL-C (mg/dL)	174 ± 43	173 ± 43	185 ± 46	176 ± 44	0.432	
Total cholesterol to HDL-C ratio	5.0 ± 1.3	5.0 ± 1.3	5.4 ± 1.3	5.2 ± 1.3	0.052	
Triglycerides (mg/dL)	179 ± 101	179 ± 101	206 ± 107	180 ± 91	**0.024**	a ≠ b
Handgrip strength (kg)	24.9 ± 5.8	25.0 ± 5.7	22.2 ± 5.5	23.0 ± 5.7	**<0.001**	a ≠ b, a ≠ c

*A p-value <0.05 indicates statistical significance*.

*Data presented as number and percentage or mean ± standard deviation. LDL-C, low-density lipoprotein cholesterol; HDL-C, high-density lipoprotein cholesterol; NAFLD, nonalcoholic fatty liver disease*.

*Chi-square test and one-way analysis of variance (ANOVA) were used to compare the baseline characteristics of study participants among the smoking status categories. Post hoc multiple comparison analysis was performed with Bonferroni correction*.

*a ≠ b ≠ c: Statistically significant difference among never smoker (a), former smoker (b), and current smoker (c)*.

*a ≠ b, a ≠ c: Statistically significant difference between never smoker (a) and former smoker (b), and between never smoker (a) and current smoker (c); however, there was no significant difference between former smoker (b) and current smoker (c)*.

*b ≠ c: Statistically significant difference between former smoker (b) and current smoker (c)*.

*a ≠ b, b ≠ c: Statistically significant difference between never smoker (a) and former smoker (b), and between former smoker (b) and current smoker (c); however, there was no significant difference between never smoker (a) and current smoker (c)*.

### All-Cause Mortality

**Figure 2:**
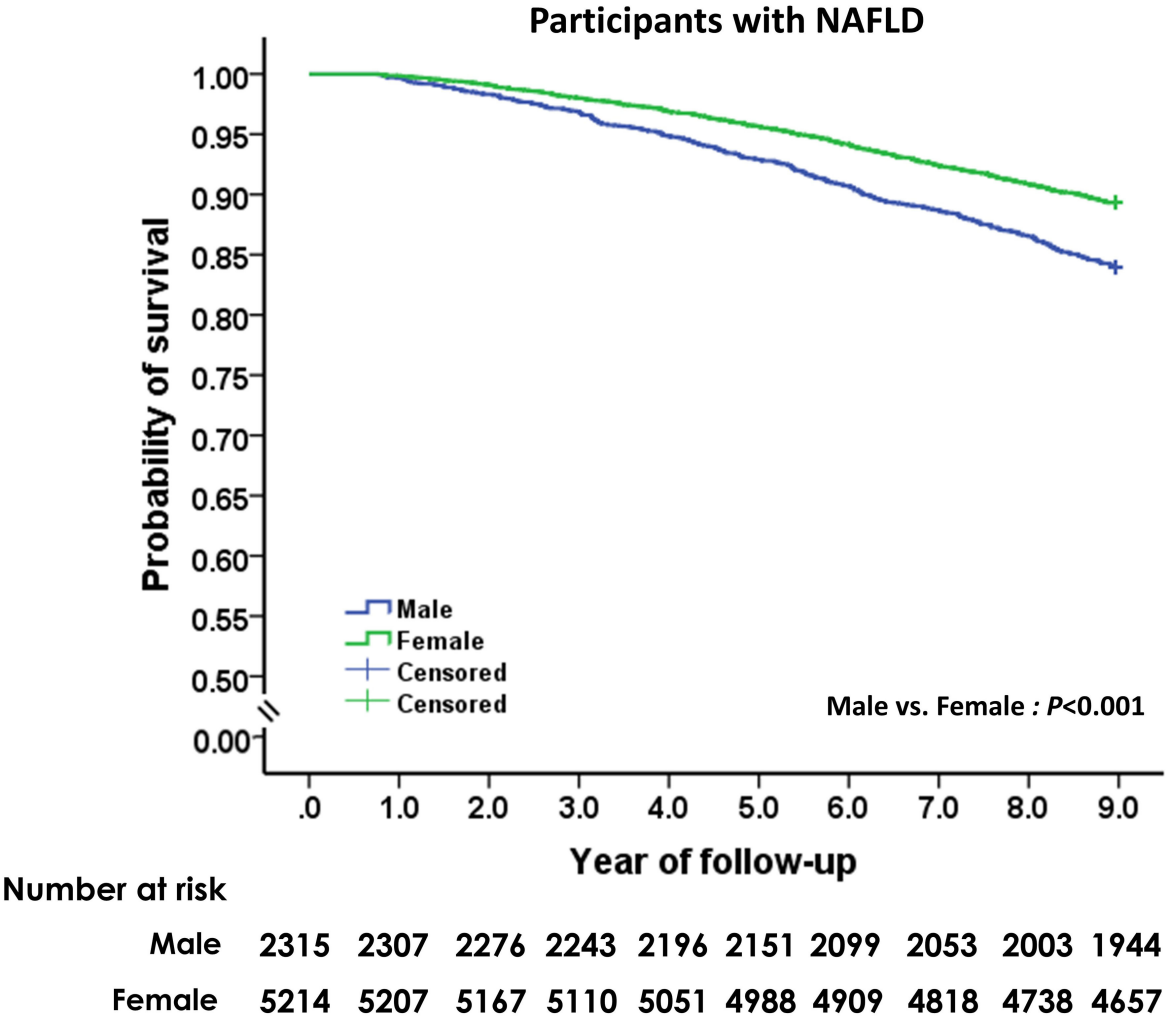
Unadjusted Kaplan-Meier survival analysis of men and women with non-alcoholic fatty liver disease.

The mean follow-up period for the study cohort was 8.52 ± 1.43 years (range: 0.76–8.96). During the 64,116 person-years of follow-up, 928 participants with NAFLD died, and the cumulative all-cause mortality was 14.5 per 1,000 person-years. As shown in Figure 2, mortality from all causes was higher among male subjects with NAFLD than female subjects with NAFLD (19.2 vs. 12.5 per 1,000 person years, HR: 1.33, 95% CI: 1.17–1.52; *p* < 0.001). Compared to subjects without fatty liver and significant alcohol use, participants with NAFLD had non-significantly lower overall mortality (HR: 0.90, 95% CI: 0.68–1.20; *p* = 0.46) after adjusting for age and sex.

### Smoking and Risk of All-Cause Mortality

**Figure 3:**
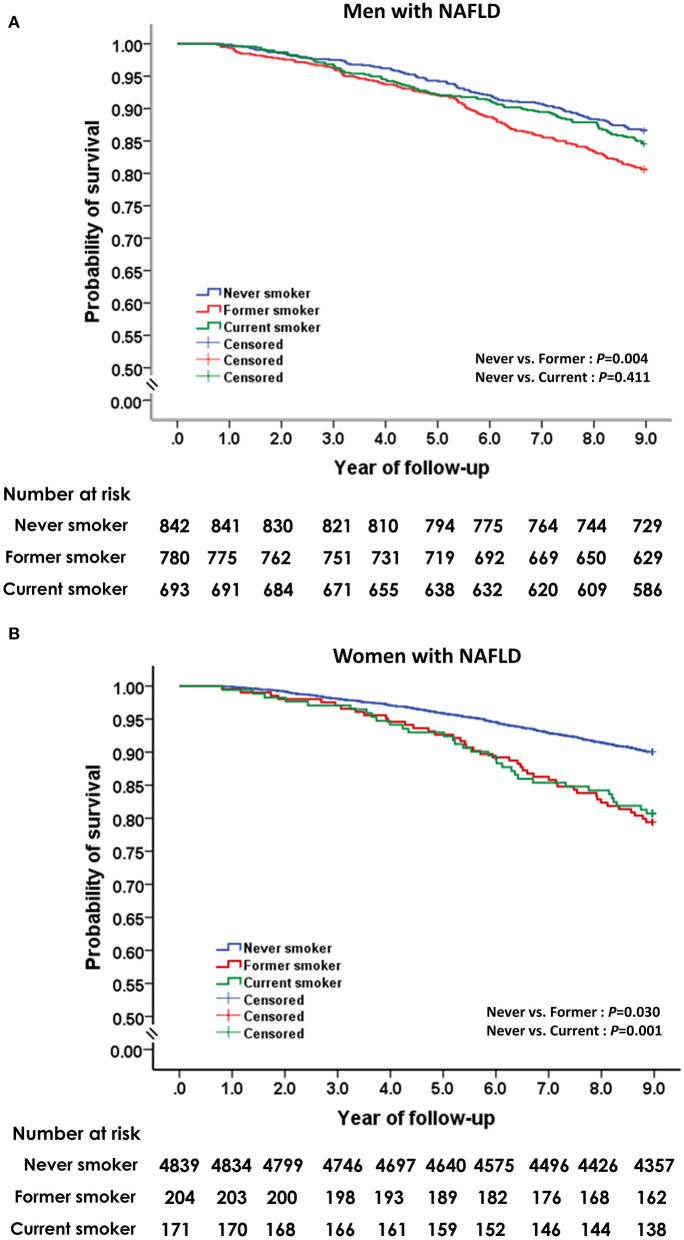
Unadjusted Kaplan-Meier survival analysis for smoking status on all-cause mortality in men **(A)** and women **(B)** with non-alcoholic fatty liver disease.

The effect of smoking status on all-cause mortality in men and women with NAFLD is depicted in Figure 3. Self-reported current smoking significantly increased the risk of death in women, but not in men with NAFLD (Table 3). For women, after adjusting for age, BMI, alcohol intake, regular exercise, diabetes, hypertension, metabolic syndrome, and history of cardiovascular disease and cerebrovascular disease (model 1), the multivariable-adjusted HR (95%CI) for all-cause mortality comparing current smoking to never smoking was 1.99 (95% CI: 1.11–3.54). For model 2, which included LDL-C, HDL-C, triglycerides, and handgrip strength to the list of variables included in model 1, the effect of current smoking on all-cause mortality in women remained significant and was slightly increased (aHR: 2.09, 95% CI: 1.18–3.71). To investigate the combined effect of smoking and alcohol consumption, we performed stratified analysis by drinking level (<10 and ≥10 gm per day). Among women who drank <10 gm per day, former or current smoking was not associated with a significant effect on mortality compared to never smokers. Among women who drank ≥10 gm per day, former smokers (aHR: 5.91, 95% CI: 2.05–17.0) and current smokers (aHR: 13.8, 95% CI: 1.66–145) had a significant increase in the risk of death after adjusting for all potential variables.

**Table 3:** Overall mortality of study participants with NAFLD by smoking status and smoking intensity.

**Variables**	**Person-years**	**Deaths**	**Deaths per 1,000 person-years**	**Age-adjusted HR (95% CI)**	**Multivariable-adjusted HR (95% CI)**	
					**Model 1**	**Model 2**
**MEN (*****n*** **=** **2,315)**						
**Smoking status**						
Never smoker (*n* = 842)	7,131.2	113	15.8	Reference	Reference	Reference
Former smoker (*n* = 780)	6,425.9	151	23.5	1.16 (0.82–1.64)	1.27 (0.90–1.79)	1.31 (0.93–1.85)
Current smoker (*n* = 693)	5,812.4	107	18.4	1.35 (0.94–1.95)	1.52 (0.98–2.33)	1.41 (0.96–2.08)
**Pack-years**						
0 (*n* = 842)	7,131.2	113	15.8	Reference	Reference	Reference
<10 (*n* = 116)	982.4	12	12.2	1.79 (0.86–3.74)	2.10 (0.90–4.88)	1.46 (0.65–3.25)
10-19.9 (*n* = 93)	805.4	11	13.7	1.50 (0.54–4.19)	1.64 (0.44–6.04)	1.73 (0.53–5.64)
≥20 (*n* = 124)	1,051.7	20	19.0	1.35 (0.72–2.52)	1.20 (0.51–2.81)	1.09 (0.53–2.23)
**WOMEN (*****n*** **=** **5,214)**						
**Smoking status**						
Never smoker (*n* = 4,839)	41,646.1	482	11.6	Reference	Reference	Reference
Former smoker (*n* = 204)	1,687.9	42	24.9	1.31 (0.80–2.15)	1.34 (0.86–2.10)	1.32 (0.84–2.11)
Current smoker (*n* = 171)	1,413.3	33	23.3	1.90 (1.13–3.20)	1.99 (1.11–3.54)	2.09 (1.18–3.71)
**Pack-years**						
0 (*n* = 4,839)	41,646.1	482	11.6	Reference	Reference	Reference
<10 (*n* = 24)	204.2	4	19.6	0.64 (0.24–1.76)	0.17 (0.03–1.10)	0.19 (0.03–1.19)
≥10 (*n* = 21)	176.9	3	17.0	4.60 (2.31–9.15)	5.25 (2.26–12.2)	5.40 (2.19–13.4)

*NAFLD, nonalcoholic fatty liver disease; HR, hazard ratio; CI, confidence interval*.

*Multivariable-adjusted model 1 was adjusted for age, body mass index, alcohol intake, regular exercise, diabetes, hypertension, metabolic syndrome, history of cardiovascular disease, and history of cerebrovascular disease*.

*Multivariable-adjusted model 2 was adjusted for the parameters in model 1 plus low-density lipoprotein cholesterol, high-density lipoprotein cholesterol, triglycerides, and handgrip strength*.

We further explored the effect of cigarette smoking on overall mortality in patients with NAFLD based on smoking intensity (Table 3). Compared to never smokers, smoking ≥10 pack-years was associated with a significantly increased risk of death in women (aHR: 5.40, 95% CI: 2.19–13.4), but not in men after adjusting for age, BMI, alcohol intake, regular exercise, comorbidities, serum lipid profiles, and handgrip strength. Smoking <10 pack-years did not show a significant effect on mortality in either women or men with NAFLD. We also assessed the effects of smoking intensity on mortality in women with NAFLD according to drinking level. Among women who drank ≥10 gm per day, smoking ≥10 pack-years strongly significantly increased the risk of death (aHR: 310, 95% CI: 78–1,296) compared to never smokers after adjusting for all potential variables. Among women who drank <10 gm per day, smoking ≥10 pack-years was not associated with a significant effect on mortality compared to never smokers (aHR: 2.31, 95% CI: 0.80–6.67).

In the multivariable-adjusted model for the mortality risk, we found significant effects of sarcopenia and metabolic features, especially diabetes, on death among men and women with NAFLD (Table 4). To investigate the association between cigarette smoking and diabetes on mortality, we performed the stratified analysis by comorbid diabetes controlling for age, BMI, alcohol intake, regular exercise, hypertension, underlying atherosclerotic diseases, serum lipid profiles, and handgrip strength (Table 5). The significantly increased risk of mortality was observed with current smoking (aHR: 2.07, 95% CI: 1.07–4.03) and smoking ≥10 pack-years (aHR: 6.82, 95% CI: 2.66–17.5) in non-diabetic women but not in men. In the diabetic subjects, current smoking significantly increased the risk of mortality compared with never smoking (aHR: 1.76, 95% CI: 1.09–2.84) in men, but showed only a trend toward harm among women (aHR: 1.61, 95% CI: 0.71–3.65). The potential hazard of smoking pack-years on mortality was observed in both men and women with diabetes but did not reach statistical significance.

**Table 4:** Effect of smoking status on all-cause mortality among men and women with NAFLD after adjusting for all variables.

	**Men**		**Women**	
	**Adjusted HR (95% CI)**	***p*-value**	**Adjusted HR (95% CI)**	***p*-value**
Smoking status				
Never smoke	Reference		Reference	
Former smoke	1.31 (0.93–1.85)	0.113	1.33 (0.84–2.11)	0.215
Current smoke	1.41 (0.96–2.08)	0.077	2.09 (1.18–3.71)	**0.014**
Age (years)	1.05 (1.03–1.07)	**<0.001**	1.04 (0.99–1.08)	0.058
Body mass index (kg/m^2^)	1.00 (0.94–1.07)	0.875	0.99 (0.93–1.05)	0.619
Alcohol intake	0.69 (0.37–1.29)	0.227	0.77 (0.29–2.09)	0.597
Regular exercise	1.44 (0.85–2.43)	0.163	0.70 (0.43–1.14)	0.143
Cerebrovascular disease	1.43 (0.55–3.67)	0.442	0.98 (0.44–2.16)	0.948
Cardiovascular disease	1.71 (0.88–3.33)	0.107	1.67 (0.89–3.14)	0.106
Hypertension	1.52 (0.93–2.50)	0.092	1.39 (1.07–1.80)	**0.018**
Diabetes	1.65 (1.13–2.41)	**0.012**	2.09 (1.43–3.04)	**0.001**
Metabolic syndrome	0.86 (0.52–1.42)	0.545	1.06 (0.75–1.49)	0.742
LDL-C (mg/dL)	1.00 (0.99–1.01)	0.052	1.00 (0.99–1.01)	0.626
HDL-C (mg/dL)	0.97 (0.94–1.01)	0.064	0.99 (0.98–1.02)	0.874
Triglycerides (mg/dL)	1.00 (0.99–1.002)	0.423	1.001 (1.0001–1.002)	**0.039**
Handgrip strength (kg)	0.95 (0.91–0.99)	**0.018**	0.96 (0.93–0.99)	**0.024**

*CI, confidence interval; LDL-C, low-density lipoprotein cholesterol; HDL-C, high-density lipoprotein cholesterol; HR, hazard ratio; NAFLD, non-alcoholic fatty liver disease*.

**Table 5:** Overall mortality of study participants with NAFLD by diabetes and smoking status.

**Variables**	**Person-years**	**Deaths**	**Deaths per 1,000 person-years**	**Age-adjusted HR (95% CI)**	**Multivariable-adjusted HR (95% CI)**	
					**Model 1**	**Model 2**
**MEN WITHOUT DIABETES (*****n*** **=** **1,894)**						
**Smoking status**						
Never smoker (*n* = 694)	5938.6	75	12.6	Reference	Reference	Reference
Former smoker (*n* = 609)	5124.8	101	19.7	1.22 (0.82–1.81)	1.29 (0.90–1.86)	1.36 (0.95–1.93)
Current smoker (*n* = 59)	5027.7	75	14.9	1.32 (0.75–2.32)	1.42 (0.83–2.42)	1.30 (0.84–2.00)
**Pack-years**						
0 (*n* = 694)	5938.6	75	12.6	Reference	Reference	Reference
<10 (*n* = 101)	858.7	9	10.5	1.92 (0.65–5.66)	2.19 (0.75–6.36)	1.38 (0.49–3.86)
>10 (*n* = 186)	1599.6	21	13.1	1.22 (0.55–2.71)	1.14 (0.41–3.15)	1.12 (0.40–3.16)
**MEN WITH DIABETES (*****n*** **=** **421)**						
**Smoking status**						
Never smoker (*n* = 148)	1192.7	38	31.9	Reference	Reference	Reference
Former smoker (*n* = 171)	1301.1	50	38.4	1.04 (0.66–1.64)	1.13 (0.72–1.77)	1.21 (0.75–1.96)
Current smoker (*n* = 102)	784.8	32	40.8	1.48 (0.67–3.28)	1.69 (0.81–3.57)	1.76 (1.09–2.84)
**Pack-years**						
0 (*n* = 148)	1192.7	38	31.9	Reference	Reference	Reference
<10 (*n* = 9)	69.9	3	42.9	1.94 (0.34–11.2)	6.07 (1.06–34.9)	9.98 (1.57–63.3)
>10 (*n* = 37)	311.3	10	32.1	1.63 (0.44–6.11)	2.32 (0.38–14.2)	2.73 (0.47–15.9)
**WOMEN WITHOUT DIABETES (*****n*** **=** **4,297)**						
**Smoking status**						
Never smoker (*n* = 3,993)	34590.4	9.89	342	Reference	Reference	Reference
Former smoker (*n* = 160)	1320.9	24.98	33.0	1.31 (0.80–2.14)	1.32 (0.78–2.21)	1.33 (0.81–2.19)
Current smoker (*n* = 144)	1204.2	19.93	24.0	2.06 (1.09–3.90)	1.96 (1.01–3.82)	2.07 (1.07–4.03)
**Pack-years**						
0 (*n* = 3,993)	34590.4	342	9.9	Reference	Reference	Reference
<10 (*n* = 20)	173.6	3	17.3	0.49 (0.17–1.45)	0.37 (0.06–1.98)	0.44 (0.08–2.31)
>10 (*n* = 16)	136.8	2	14.6	6.11 (2.85–13.0)	6.48 (2.89–14.6)	6.84 (2.71–17.2)
**WOMEN WITH DIABETES (*****n*** **=** **917)**						
**Smoking status**						
Never smoker (*n* = 846)	7055.8	19.84	140	Reference	Reference	Reference
Former smoker (*n* = 44)	366.9	24.53	9.0	1.46 (0.69–3.09)	1.56 (0.75–3.27)	1.54 (0.73–3.26)
Current smoker (*n* = 27)	209.1	43.05	9.0	1.81 (0.93–3.52)	1.78 (0.71–4.50)	1.61 (0.71–3.65)
**Pack-years**						
0 (*n* = 846)	7055.8	140	19.8	Reference	Reference	Reference
<10 (*n* = 4)	30.6	1	32.7	0.66 (0.27–1.58)	NA	NA
>10 (*n* = 5)	40.1	1	24.9	1.70 (0.18–15.7)	2.23 (0.26–19.0)	1.86 (0.37–9.33)

*NA, not assessed; NAFLD, nonalcoholic fatty liver disease; HR, hazard ratio; CI, confidence interval*.

*Multivariable-adjusted model 1 was adjusted for age, body mass index, alcohol intake, regular exercise, hypertension, history of cardiovascular disease, and history of cerebrovascular disease*.

*Multivariable-adjusted model 2 was adjusted for the parameters in model 1 plus low-density lipoprotein cholesterol, high-density lipoprotein cholesterol, triglycerides, and handgrip strength*.

## Discussion

Though there is evidence suggesting potential detrimental effects of cigarette smoking in patients with NAFLD, the association between cigarette smoking and mortality has not been adequately evaluated in this population. In this nationally representative sample of 7,529 adults with NAFLD, we demonstrate that current smoking is associated with a robust and significant increase in all-cause mortality among women with NAFLD. Furthermore, smoking ≥10 pack-years is associated with an increased risk of mortality in women with NAFLD. We failed to find a significantly harmful effect of smoking in men with NAFLD. Interestingly, we found that modest alcohol consumption has a synergistic effect with cigarette smoking on all-cause mortality in women with NAFLD.

It has been suggested that cigarette smoking reduces life expectancy, primarily by increasing the risks of chronic obstructive pulmonary disease, cancer, and cardiovascular disease (10). However, epidemiological data specific to the relationship between cigarette smoking and the risk of death in NAFLD is scarce. Our study revealed current smoking to be significantly associated with a 2-fold increased risk of all-cause mortality among women with NAFLD, independent of alcohol intake, exercise, diabetes, hypertension, metabolic syndrome, underlying atherosclerotic diseases, lipid profiles, and handgrip strength. These findings are virtually identical to those from studies that evaluated the contemporary risks of smoking in Western and Asian countries, where the relative risk of all-cause mortality in current smokers compared to never smokers has been consistently reported to be 2.0 to 3.0 (24–26). Importantly, our findings indicate an elevated risk of mortality in patients with NAFLD based on the cumulative quantity of cigarettes smoked. In particular, women with NAFLD who smoked ≥10 pack-years had approximately 5-fold increased overall mortality compared to never smokers. This finding was adjusted for a range of potential confounders, including BMI, alcohol intake, exercise, and several comorbidities.

The influence of alcohol consumption within the accepted safe limits on the association between cigarette smoking and the risk of death by any cause was investigated in our NAFLD study. That analysis revealed an increased risk of all-cause mortality among women who smoke and drink a moderate amount alcohol, defined as consumption of equal to or more than 10 g per day. This finding is consistent with prospective studies that found even modest alcohol intake to be associated with possible disease progression (27), and more significantly, with cancer development in patients with NAFLD (28).

Gender-related differences in the effects of cigarette smoking on the risk of death are not well-established. In this NAFLD cohort, we found smoking to be more harmful in women than in men, with a larger negative impact from the same number of smoking pack-years. Since patients with NAFLD are at higher risk for cardiovascular disease compared to general population (4), active smokers with NAFLD are more likely to die from cardiovascular disease. This assertion is supported by evidence from a Danish study that showed higher cardiovascular disease risk in women who smoke than in men who smoke (29). This may be due to the fact that smoking causes downregulation of estrogen-dependent vasodilatation of the endothelial wall (30). Hence, the effect of cigarette smoking on the risk of atherothrombotic clinical events was higher in postmenopausal women (31). Cigarette smoking was also found to be a risk factor for developing extrahepatic malignancies (7). This association is clinically relevant since de novo tumors are a leading cause of death among NAFLD patients (3, 5, 6). Overall, these data indicate that female smokers with NAFLD are at higher risk for smoking-related morbidity and mortality. However, the effect of smoking on disease-specific mortality was not analyzed in our population because the NHES database could not capture the causes of death.

Although the prevalence of current smoking was somewhat higher among men than among women, we observed no significantly increased risk for overall mortality among men who currently smoke compared with never smokers. Further, we did not find a significant association between smoking ≥10 pack-years and mortality over the 9-year period among men with NAFLD. It is plausible that smoking-mediated diseases occur at a prolonged smoking duration and higher number of cigarettes smoked in male subjects, and it may require coincident genetic predisposition. Smoking cessation almost completely reverses the risk of cardiovascular disease due to smoking, which makes it potentially the most effective and lifesaving intervention available for those at risk for and those with existing cardiovascular disease (32). Unfortunately, we observed a non-significant increase in overall mortality among those who stopped smoking at an older age (the mean age of 55 years or older in both genders). The potentially higher risk of death in former smokers compared with never smokers might be explained by a tendency for smokers, particularly older smokers, to quit due to ill health.

Although the mechanisms by which smoking contributes to mortality among NAFLD patients remain unknown, it has been shown that smoking would accelerate atherosclerosis by inducing insulin resistance and altering lipid metabolism (33–35). However, we found that after adjusting for metabolic parameters (diabetes, hypertension, and lipid profiles), the association between current smoking and mortality risk remained significant, indicating that atherogenic risk factors did not fully explain the relationship. Another potential mechanism is that tobacco constituents induce or promote oxidative stress/inflammatory pathways, which can play a role in the pathogenesis of NAFLD (36, 37). Recently, a longitudinal cohort study demonstrated a dose-response relationship of smoking with the development and progression of NAFLD (38). Hence, it is anticipated that increased risk of steatohepatitis and advanced fibrosis possibly driven by smoking in patients with NAFLD may significantly impact their mortality. However, the NHES did not collect variables for estimating non-invasive fibrosis scores (e.g., Hepamet, Fibrosis-4, and NAFLD fibrosis scores) to identifying individuals in the NAFLD population at risk for advanced fibrosis. Given that diabetes is a major contributor to perpetual chronic injury leading to eventual steatohepatitis, advanced liver fibrosis, and hepatocellular carcinoma (39), individuals with diabetes may represent the target population with a high-risk of advanced liver disease. Thus, NAFLD patients with diabetes who continue smoking should be a subgroup with high mortality risk. Supporting this assumption, our analysis showed that current smokers with diabetes, particularly men, had a significantly greater probability of deaths than those who did not smoke. Nevertheless, a low number of current smokers in the diabetes subgroups limit the power to estimate smoking status and intensity associated with mortality, especially in women.

Sarcopenia that is defined as the loss of skeletal muscle mass and strength has been increasingly recognized in patients with NAFLD. It is a crucial indicator of adverse outcomes in patients with cirrhosis, including hepatic decompensation and premature mortality (40). In the Cox regression model, we found that weaker handgrip strength was significantly associated with an increased risk of death among NAFLD population. Thus, it may be suggested that the substantially increased risk of death may be mediated via sarcopenia among smoking women with NAFLD, who were more likely to have low measurements of handgrip strength and BMI. This finding is supported by data from a meta-analysis involving 22,515 participants reporting that cigarette smoking may contribute to the development of sarcopenia (41). Physiological mechanisms are complex, and several studies showed that cigarette smoking induced muscle protein degradation via oxidative stress and chronic inflammation (42).

This study has some limitations. Firstly, the diagnosis of NAFLD was made using LAP and not by gold standard tissue biopsy. Although a validation study showed that LAP exhibited high diagnostic accuracy for identifying NAFLD (23), some of our study participants would inevitably have been misclassified as NAFLD. Using a model based on anthropomorphic and laboratory data enabled us to study a large population-based sample and facilitate avoidance of ascertainment bias that is routinely found in clinical studies of conveniently selected patients. Secondly, the smoking assessment was based on self-reports and was not verified by objective measures of smoking (urinary cotinine and breath carbon monoxide tests). The assessment of smoking status by questionnaires may lead to inaccurate measures of smoking exposure due to smoking denial or difficulty in recalling the quantity and duration of smoking (43). This misclassification potentially leads to the underestimation of the detrimental effects of smoking exposure. However, nationwide studies in Canada and the United States revealed that smoking behavior self-reported by the participants in such surveys was highly consistent with estimates based on urinary or serum cotinine concentration (44–46). This data indicates that a low-cost questionnaire survey applicable to large samples can effectively determine the smoking status of the general population. Lastly, the lack of a significant difference in mortality between the general population with and without NAFLD in this study should not prove that NAFLD does not lead to overall mortality. This contrasts with the findings from previous studies frequently conducted in NAFLD patients who underwent liver biopsies at specialty liver clinics (5, 6, 17). The difference between those and population-based studies is probably attributable to selection bias entailed in referral patients. Instead, we believe that it is likely a type II error, given a low number of deaths during a 9-year follow-up in our population-based sample. The study of the impact of NAFLD on mortality may require a much longer follow-up to draw a firm conclusion.

## Conclusion

The results from this nationwide population-based cohort study suggest a detrimental effect of cigarette smoking on all-cause mortality, with a similar but more definite association in women than in men with NAFLD. In addition, our findings are consistent with previous studies showing a relationship between smoking intensity and mortality, where an increase in the quantification of cigarette smoke becomes more harmful. The results of this study can be used to improve counseling of the growing population of patients with NAFLD by discouraging smoking.

## Data Availability Statement

The raw data supporting the conclusions of this article will be made available by the authors, without undue reservation.

## Ethics Statement

The studies involving human participants were reviewed and approved by the Ethics Committee of the Faculty of Medicine Siriraj Hospital, Mahidol University, Bangkok, Thailand (COA no. Si 513/2019). The patients/participants provided their written informed consent to participate in this study.

## Author Contributions

PC and WA contributed to study concept and design. PC drafted the initial manuscript. PC, KK, and WA contributed to analyze and interpret data, and approved the final version of the manuscript. All authors contributed to the article and approved the submitted version.

## Conflict of Interest

The authors declare that the research was conducted in the absence of any commercial or financial relationships that could be construed as a potential conflict of interest.
